# Efficacy of music-based telerehabilitation after stroke: protocol description of a randomised controlled trial

**DOI:** 10.1136/bmjopen-2026-124110

**Published:** 2026-09-24

**Authors:** Ella Laakso, Emma Oksanen, Lotta Maaria Kaila, Ilja Salakka, Jaakko Kauramäki, Netta Lahdenperä, Anni Pitkäniemi, Aleksi J Sihvonen, Nella Moisseinen, Teppo Särkämö

**Affiliations:** 1 Cognitive Brain Research Unit and Centre of Excellence in Music, Mind, Body and Brain - Department of Psychology, Faculty of Medicine, University of Helsinki, Helsinki, Finland; 2 Department of Neurology, HUS Helsinki University Hospital, Helsinki, Finland; 3 Queensland Aphasia Research Centre, The University of Queensland, Brisbane, Queensland, Australia

**Keywords:** Stroke, Cognition, Stroke Rehabilitation, Telemedicine, Clinical trials

## Abstract

**Introduction:**

With the rapid ageing of the population, the prevalence and public health burden of stroke continue to rise. Many patients are left without sufficient access to multidisciplinary rehabilitation that addresses the complexity of stroke symptoms, including deficits in motor, cognitive and verbal functioning as well as emotional well-being. Several music-based rehabilitation methods, such as instrument playing, rhythm exercises, singing and music listening, have shown promising effects across these domains. In addition, interest in stroke telerehabilitation has grown as a way to improve accessibility and efficiency of rehabilitation when healthcare resources are limited. However, the effectiveness and usability of a multidimensional, music-based telerehabilitation programme for stroke recovery have not yet been systematically investigated. In the present project, we (1) developed a novel music-based telerehabilitation programme for patients who had a stroke, which combines different music-based rehabilitation methods (singing, playing, rhythmic movement, music listening); (2) evaluate whether the programme can enhance verbal, motor, cognitive and emotional recovery and induce structural and functional neuroplasticity changes in patients who had a stroke; and (3) explore how patients’ clinical and demographic factors, musical background and current post-stroke musical skills affect the programme’s efficacy.

**Methods and analysis:**

In this parallel-group randomised controlled trial, 100 patients who had a stroke recruited from Southern Finland receive either the music-based telerehabilitation programme (10 weeks, 5 days/week, 60 min/day) or standard care and rehabilitation. Participants in the music intervention group are assigned according to lesion laterality either to singing-based (left-hemisphere stroke) or playing-based (right-hemisphere stroke) training. Both subgroups receive rhythm-based training targeting cognitive deficits and music listening targeting emotional well-being. Efficacy is assessed over a 6-month follow-up period using standardised verbal, motor and neuropsychological tests; questionnaires on mood, daily functioning and quality of life; and structural and functional MRI. Clinical and demographic background information as well as musical abilities are assessed at baseline.

**Ethics and dissemination:**

Ethical approval has been obtained in Finland 12/24. Results will be presented in international and national conferences and published in international scientific journals and in journals for clinical professionals and patient associations in Finland.

**Trial registration number:**

NCT06804304.

**STRENGTHS AND LIMITATIONS OF THIS STUDY**

- The randomised controlled trial design of this study strengthens internal validity and allows for causal inference regarding the effectiveness of this multidimensional music-based telerehabilitation programme that integrates multiple music-based rehabilitation methods into a single platform.
- A sufficiently large sample size increases the statistical power and generalisability within the target population.
- The comprehensive set of outcome measures offers a detailed and holistic view on the participant’s verbal, motor, cognitive, emotional and neural recovery.
- Patient and public involvement has been included throughout the planning and piloting stage of the study to ensure meaningful intervention content and clinical relevance of the outcome measures.
- Selection bias may affect the sample and results, as patients who are willing to participate in a 10-week, 5-day-per-week telerehabilitation programme may differ systematically from the broader stroke population (eg, higher motivation, better baseline functioning, better digital literacy).

## Introduction

### Background

 Stroke is a severe neurological condition where blood flow to the brain is disrupted due to a blocked or ruptured blood vessel, resulting in cerebral damage and loss of functioning. In 2021, there were approximately 12 million new cases and 94 million prevalent cases of stroke globally,^1^ and stroke was one of the top causes for disability-adjusted life-years after COVID-19, ischaemic heart disease and neonatal disorders.^2^ Globally, stroke incurs substantial economic costs, both for its direct (US$338 billion^3^) and indirect (US$271 billion^4^) care. While the age-adjusted incidence and prevalence of stroke have decreased during the last decades, the rapid ageing of the global population will lead to a substantial increase in the absolute number of patients with stroke and associated socioeconomic burden in the near future.^1^

The neurological impairments caused by stroke are diverse; the most common deficits are in motor functions (hemiparesis: 80%^5^), cognitive functions (deficits in, eg, attention, memory and executive functions: 50–60%^6 7^) and verbal functions (aphasia: 30–40%^8^). Furthermore, post-stroke fatigue (50%^9 10^) and mood disturbances, such as depression (30%^11^) are common after stroke. Given the high co-occurrence of these deficits, patients often benefit from multidisciplinary rehabilitation.^12 13^ Accurate estimates of access to multidisciplinary stroke rehabilitation are difficult to establish since rehabilitation services both within and between countries and healthcare systems vary significantly. Nevertheless, studies have identified unmet needs related to stroke rehabilitation, with information, rehabilitation and physical functioning being among the most commonly reported areas of unmet needs.^14 15^ Therefore, new cost-effective rehabilitation methods are needed to increase availability and accessibility especially for patients who are not offered sufficient rehabilitation in public healthcare, cannot afford private rehabilitation services or live in areas where access to rehabilitation is limited. These considerations motivate the implementation of versatile and effective rehabilitation approaches, such as music-based interventions, to support long-term recovery and complement conventional rehabilitation.

Music, an inherently meaningful and pleasurable component of everyday human experience, can provide comfort and sense of familiarity after a sudden adverse event such as stroke, but also serve as an effective and motivating tool in neurological rehabilitation. Music-based interventions for patients who had a stroke have previously been studied in a number of randomised controlled trials (RCTs) and brain imaging studies (for meta-analysis and reviews see studies by Popescu *et al*, Sihvonen *et al* and Wang *et al*^16–18^). These interventions use the intrinsic ability of music to enhance mood, arousal and motivation as well as to activate widespread neural networks and support neuroplasticity in spared brain regions.^17 19^

Previous research demonstrates that music-based motor rehabilitation methods using musical instruments, such as music-supported therapy (MST), can improve upper-extremity gross and fine movements, attention, executive functions and mood,^17 20 21^ while promoting neural activity and connectivity between motor and auditory regions.^22 23^ Similarly, interventions using rhythm to guide movement, such as rhythmic auditory stimulation (RAS), can improve motor functions, such as gait, balance and gross motor upper-extremity movements, as well as working memory in patients who had a hemiparetic stroke.^24–27^

Music-based rehabilitation methods have also been studied with respect to post-stroke aphasia. Singing-based interventions, such as melodic intonation therapy (MIT), which use the often spared ability to sing after stroke and the musical elements of speech (for description of MIT protocol, see Singing-based training), have shown to improve responsive speech production (eg, repetition, naming), spontaneous speech and communication.^16 17 28–30^ In addition, singing-based rehabilitation is linked to neuroplastic changes in the structure, neural activity and connectivity of frontotemporal language regions.^28 31 32^ Furthermore, daily listening to music, especially vocal music, during the subacute phase has shown to improve recovery of verbal memory, attention and language skills as well as emotional well-being,^17 33–36^ coupled with structural and functional neuroplastic changes in frontolimbic regions and language network.^17 36–40^

Although previous studies provide evidence for the efficacy of various music-based rehabilitation methods for stroke, each approach has thus far been studied independently and with relatively narrow rehabilitation targets, focusing either on motor, verbal, cognitive or emotional functioning. Given that post-stroke impairments are diverse in nature and often co-occur within the same patients, there is a need to develop and empirically evaluate integrated music-based rehabilitation approaches that effectively combine different music rehabilitation methods to address the varied post-stroke deficits.

As healthcare systems continue to evolve and adopt digital solutions, interest in cost-effective, scalable and accessible digital rehabilitation methods has steadily increased.^41 42^ Previous literature suggests that digital approaches may offer certain advantages, such as the potential to improve access to rehabilitation, increase training intensity and enable individual tailoring of rehabilitation content.^43–46^ At the same time, telerehabilitation is still a developing field and the use of digital tools may involve practical challenges for some patients, particularly those with aphasia, executive deficits, mobility limitations or varying levels of digital skills.^44 47^ These considerations highlight the importance of designing accessible and user-friendly telerehabilitation platforms.

In stroke rehabilitation, meta-analyses have reported comparable effects of telerehabilitation on motor, linguistic and cognitive functioning as well as on mood and quality of life to those achieved with conventional face-to-face rehabilitation.^48–53^ However, the utilisation of music in stroke telerehabilitation still remains largely unexplored. While some recent studies have reported benefits of music-assisted motor training^54–57^ and MST^58^ in telerehabilitation settings, evidence for their clinical efficacy in stroke rehabilitation remains very limited. More importantly, there are currently no telerehabilitation tools available that combine the different music-based training components (playing, singing, rhythmic movement, music listening) in a fully integrated, multicomponent music-based telerehabilitation programme that targets multiple post-stroke deficits simultaneously.

### Objectives and research questions

The objectives of this research project are to develop a new music-based multicomponent telerehabilitation programme for patients who had a stroke that combines multiple music-based rehabilitation methods (singing, instrument playing, moving to music and music listening), which have previously shown promise, and to evaluate the programme’s efficacy compared with standard care in an RCT with 100 patients who had a stroke. We aim to answer the following research questions: (1) does the music-based multicomponent telerehabilitation programme improve verbal, motor, cognitive and emotional recovery after stroke; (2) what are the structural and functional neural mechanisms underlying the programme’s rehabilitative efficacy; and (3) how patients’ clinical factors, demographic and musical background and current post-stroke musical skills affect the programme’s efficacy. We hypothesise that participants in the intervention group will show improvements in verbal, motor and cognitive functions as well as emotional well-being compared with the control group at the 3-month and the 6-month follow-up points.

## Methods and analysis

### Design

In this two-arm parallel-group RCT, 100 patients who had a stroke are randomised into two groups, a music intervention group (n=50) and a control group (n=50) and followed for 6 months (figure 1). The intervention group and the control group are further divided into left-hemisphere (n=25) and right-hemisphere stroke (n=25) subgroups. The outcome measurements are performed at baseline (pre-intervention, T1), at the 3-month follow-up (post-intervention, T2) and at the 6-month follow-up (T3). Participants in the intervention group begin the 10-week intervention within 1 week of the baseline measurement, while the control group continues to receive their usual healthcare for the duration of the follow-up period. Participants in the control group are given access to the intervention platform after the 6-month follow-up period.

**Figure 1:**
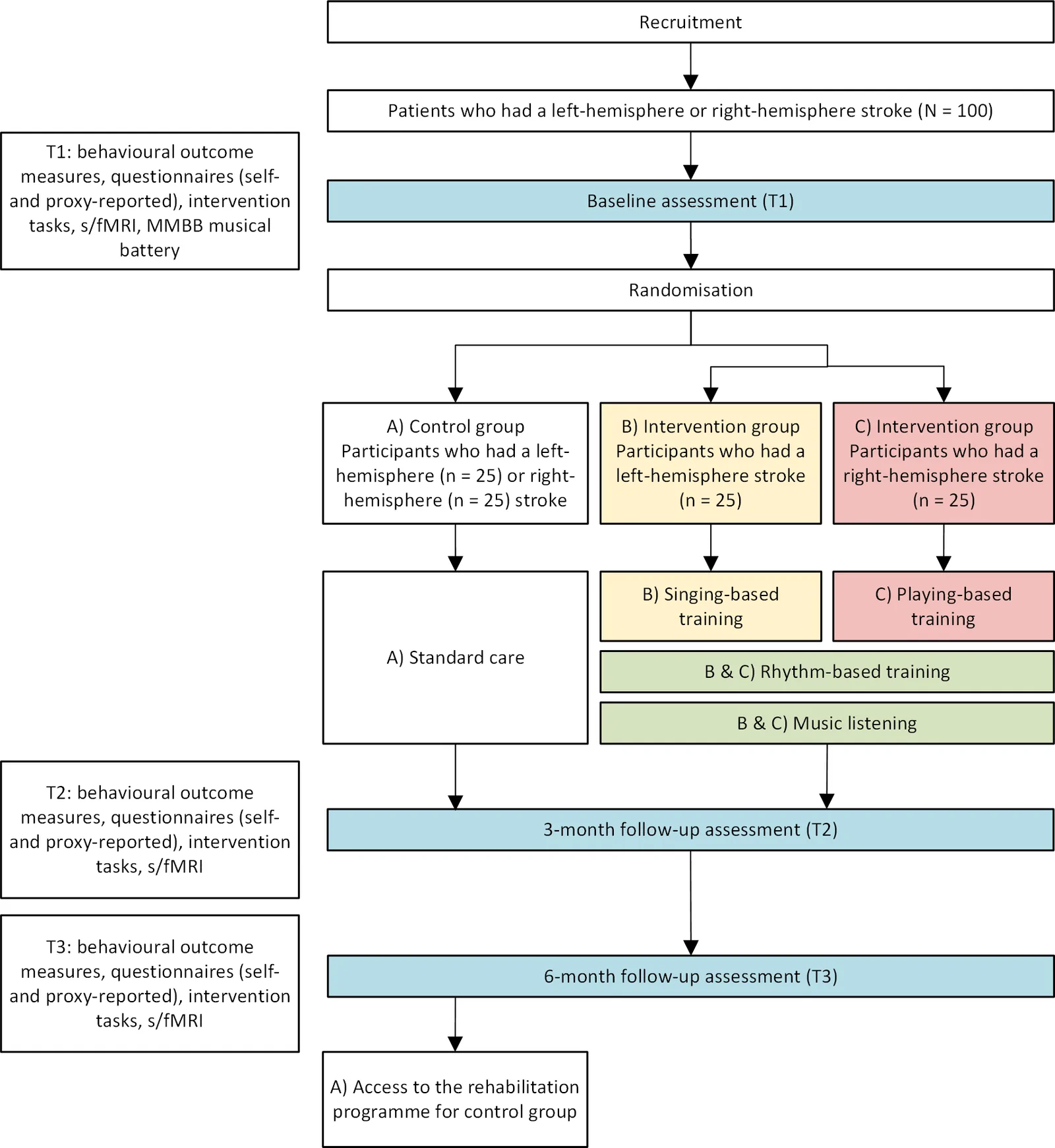
Flowchart of the randomised controlled trial. MMBB, Centre of Excellence in Music, Mind, Body and Brain; s/fMRI, structural and functional MRI.

The data collection started in April 2025 and participant recruitment is estimated to continue until December 2027. We estimate that the baseline measurements will be completed in the beginning of 2028 and the 6-month follow-up measurements in summer 2028. Data preprocessing will be carried out throughout the data collection. Final group-level statistical analyses and reporting of results are expected to take place during 2028–2029.

### Participants

#### Participant recruitment, eligibility criteria and consent

Patients who had a stroke are recruited in the Southern Finland region in cooperation with well-being services counties and patient organisations. The participants are recruited via recruitment events, flyers, social media and directly through healthcare professionals. Eligible participants meet the following criteria:

- Left-hemisphere (n=50) or right-hemisphere (n=50) stroke.
- At least 3 months post-stroke.
- Cognitive impairment (attention, memory, executive functions) combined with either language impairment (aphasia, in left-hemisphere subgroup) or motor impairment (upper-extremity hemiparesis, in right-hemisphere subgroup) caused by stroke. Impairments are reported by participant, family member, caregiver or healthcare professional.
- Age 30–80 years.
- Finnish-speaking (or bilingual, able to participate in Finnish).
- No prior significant hearing or visual impairment, severe psychiatric illness (eg, schizophrenia, bipolar disorder, severe depression) or neurological illness. Participants with prior transient ischaemic attack or minor stroke can be included, provided that they have no related persistent symptoms. Otherwise, cases of recurrent stroke are excluded.
- Able to understand the purpose of the study and provide written informed consent; if providing written consent is not possible due to the severity of motor or language impairment, consent may be expressed verbally and verified by a written confirmation by a family member or caregiver.

No previous musical expertise or education is required for participation. The trial coordinator confirms participant eligibility and gives information about the study during a telephone call. A family member or caregiver can be present during the call. After the recruitment call, all eligible participants receive an information letter about the study. After participants and, where applicable, their family member or caregiver have received full information about the study and have had the opportunity to ask questions, informed consent to participate is obtained from both the participant and the family member or caregiver. Participants may withdraw their consent and discontinue participation at any time during the study without any consequences and without providing a reason. Unless otherwise requested, the data collected up to the point of withdrawal will be retained and included in the analysis.

#### Randomisation, group allocation, and masking

Randomisation is performed by the trial coordinator after the baseline measurement, once the current severity level of the post-stroke deficits is ascertained using the National Institutes of Health Stroke Scale (NIHSS).^59^ The participants are stratified into subgroups according to lesion hemisphere (left/right), time since stroke (3–6 months/7–12 months/≥13 months) and stroke severity (NIHSS score 1–4/≥5). Randomisation is conducted within each of these strata to ensure balanced allocation across clinically relevant factors. Within each stratum, participants are randomised using a permuted block design with block sizes ranging from 1+1 to 2+2. Randomisation sequences, covering up to 50 participants per list, were generated for each stratum in advance using random.org, first for block sizes and then for allocation order. As new participants are enrolled, they are first assigned to the corresponding stratum based on the lesion hemisphere, time from stroke and the NIHSS score. Each participant is then allocated to the next available slot in that subgroup’s pregenerated randomisation sequence.

The two assessors responsible for the outcome measurements are masked to the group allocation. The participants are reminded at multiple time points not to reveal their group allocation to the assessors. If a participant’s allocation is unintentionally unmasked to the assessor during the follow-up period, the other assessor, who remains masked, will conduct the rest of the measurements.

#### Sample size and test power

Assuming that the effect of the intervention is at least medium-sized (Cohen’s d=0.6), consistent with the average effect sizes reported in previous studies of individual music-based rehabilitation methods (RAS, MST, MIT, music listening) across cognitive, motor, language and mood outcomes,^17^ a sample size calculation conducted using the G*Power software^60^ indicates that 90 participants who had a stroke are needed to achieve adequate power. This calculation is based on an effect size of d=0.6, a significance level of α=0.05 and a statistical power of 0.80. Accounting for an anticipated 10% drop-out rate, the target sample size for the study is 100 participants, with equal representation from left-hemisphere and right-hemisphere stroke (n=50 per side).

### Intervention

In the planning and preparation stage of the project (2023–2024), a novel digital music-based telerehabilitation programme for patients who had a stroke was created at the University of Helsinki. It was designed with a team consisting of researchers, clinical professionals in stroke care, music therapists and musicians, and further improved by collaborating with patients who had a stroke and healthy older-aged adults (see Patient and public involvement). The programme is broadly targeted at rehabilitation of post-stroke verbal, motor and cognitive deficits and improvement of emotional well-being. The programme uses various music-based rehabilitation methods (singing, playing, moving to the rhythm of music, music listening), which have previous promising evidence in stroke rehabilitation (for meta-analysis and reviews see studies by Popescu *et al*, Sihvonen *et al* and Wang *et al*^16–18^). The programme is designed to be engaging, motivating and user-friendly for the participants.

#### Structure and flow of the music intervention

The music-based telerehabilitation programme comprises in total 449 training and nine instruction videos. The content is organised into four training modules (figure 2); one targeted to each intervention group, and two modules common to both groups. In this intervention programme, the singing-based training module is assigned to participants with left-hemisphere stroke and the playing-based training module to participants with right-hemisphere stroke to prioritise the most prominent and functionally relevant impairments in each group. Both groups receive rhythm-based training and music listening modules which target cognitive and emotional deficits common to both groups. Overall, this multicomponent design was adopted to support participant motivation and engagement, balancing between versatility in the type of exercises and targeting of domains most affected by the stroke. The modules are described in more detail below.

**Figure 2:**
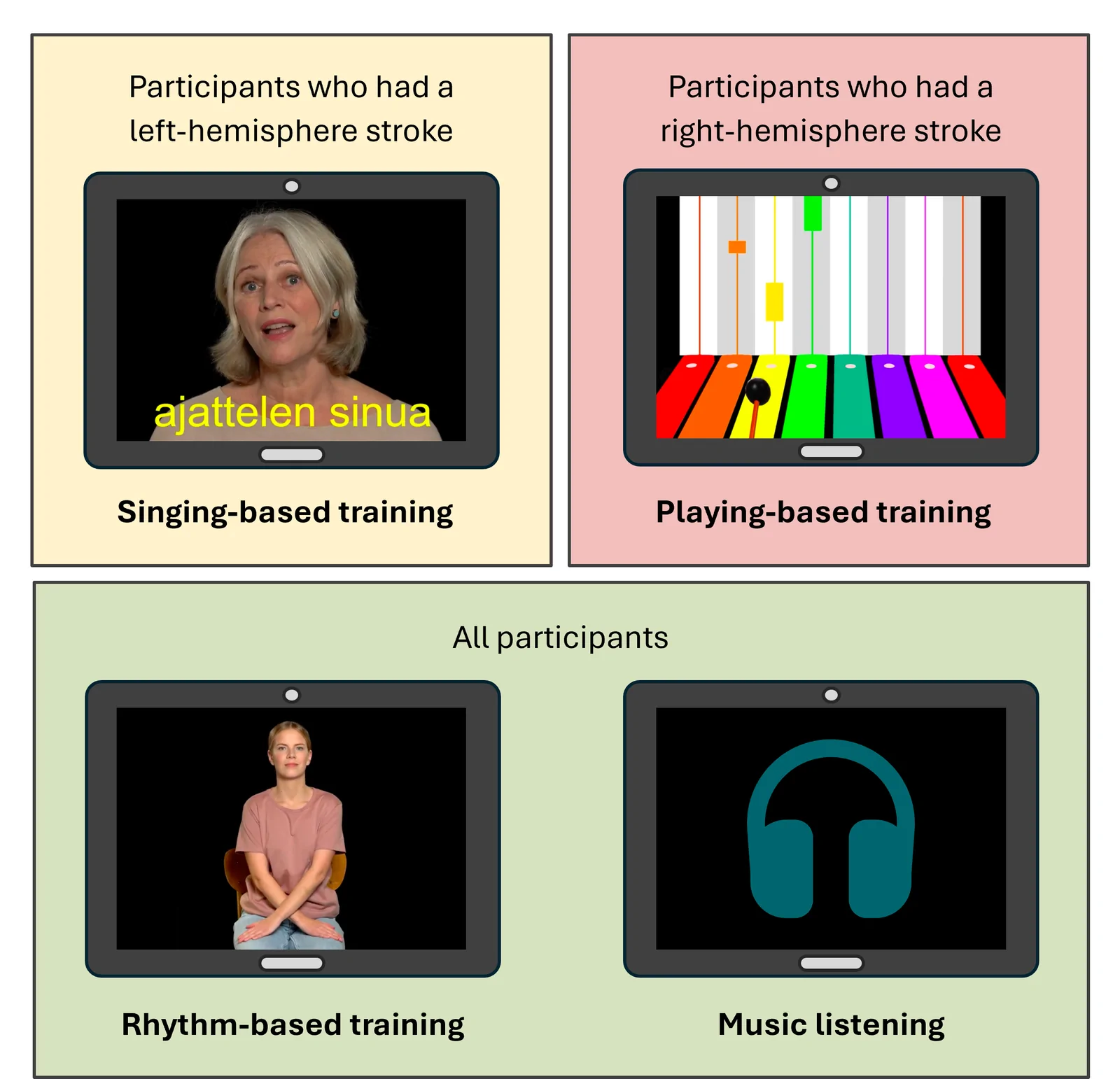
Training modules. The image contains examples of what is shown on the tablet screen during the intervention tasks.

Each training video is 3–5 min long and features a music therapist or physiotherapist who instructs participants and demonstrates the target exercise, which participants follow on a tablet computer. The music material in the playing-based and singing-based training and music listening modules comprises 20 novel songs, which were created by professional musicians and music therapists specifically for the intervention programme. Song accompaniments are played on band instruments, and the songs represent various genres to ensure diversity and allow all participants to find pleasant music. The lyrics are specifically tailored for persons with aphasia (see Patient and public involvement). The songs in the rhythm-based training module are also novel and feature clear, strong beats to facilitate entrainment of movements to the rhythm. The training videos are available at varying levels of difficulty and tempo to address the individual needs of participants and to enable progression from less to more demanding exercises.

#### Singing-based training

The primary rehabilitation target in the singing-based training module is speech production and secondary targets are attention, working memory, executive functions and emotional well-being. The module is based on previous singing-based rehabilitation methods, such as MIT and group singing interventions, which have previously shown promise in speech rehabilitation after stroke.^16 28–30^

There are two stages in this intervention module. In stage 1, participants train speech production by singing the intervention songs. The melodies and lyrics are simple, and the lyrics include themes and target words that are important and relevant to the lives of patients who had a stroke, based on findings from previous studies^61^ and feedback collected from persons with aphasia (see Patient and public involvement). In the training videos (80 videos), participants see a model singer and lyrics synchronised to the background song, as in karaoke. The training videos are available both in the typical female and male vocal range and in two different levels of tempo (slowed and original). This design enables participants to (1) follow the auditory (vocals) and motor (mouth movements) cues of the model singer to activate the mirror neuron system,^62^ (2) anticipate and prepare for the production of the next word and (3) experience singing together with the model singer and the band. This design allows for simple and intuitive training that is suitable for all.

Stage 2 of the module includes a practice protocol from MIT. The participants practise the production of formulaic phrases with the help of training videos. The phrases were extracted from lyrics of the intervention songs (four to five phrases per song, one phrase per video, altogether 99 videos). The phrases include words and sentences commonly used in everyday communication, such as ‘Hi and goodbye’ and ‘How are you?’. In each video, a music therapist demonstrates the exercise that follows the commonly used five-stage MIT protocol^63^: (1) therapist introduces the phrase by singing while participant listens; (2) therapist and participant intone (sing) the phrase together; (3) therapist and participant begin by intoning the phrase together, after which therapist fades out midway through the phrase, and participant continues independently; (4) participant immediately repeats the phrase after therapist; and (5) participant repeats the phrase after a brief delay. These five stages are repeated first by singing, then with rhythmic speech and finally with normal speech prosody, thus progressing from singing to speech. Following the MIT protocol, the verbal production is also coupled with rhythmic tapping with the non-paretic hand to time the production of syllables.

#### Playing-based training

The primary rehabilitation targets in the playing-based training module are upper-extremity gross and fine motor functions. Secondary targets include attention (including spatial attention, which is specifically impaired in participants with unilateral spatial neglect), working memory, executive functions and emotional well-being. The module is based on the MST model, which has previously shown promise in stroke rehabilitation.^17 20 21^ For training, participants are provided with a metallophone with eight metal plates in different colours tuned to C major scale and a mallet. Playing position and optimal grip are estimated by a music therapist with a background in physiotherapy. If required, participants are provided with a mallet designed for use with the hemiparetic hand. Commonly used in neurological music therapy, the metallophone is easy to play, and it has a soft, pleasurable sound.

During training, participants play the melodies of the intervention songs on the metallophone. In the training videos, participants see an on-screen replica of the metallophone plates and a mallet at the bottom of the tablet screen. The mallet demonstrates the required playing movements. In the upper half of the screen, the notes to be played are presented as descending coloured blocks, matching the colour of the target metallophone plates (see figure 2). This design enables participants to (1) anticipate the next note and play the correct plate as the block hits the plate on the screen; (2) follow the model mallet movements; and (3) experience playing together with the band. The training videos include three difficulty levels (defined by the number of notes) and two tempo levels (slowed and original) (altogether 120 videos). This allows the participants to progress and find a suitable difficulty level.

#### Rhythm-based training

The primary rehabilitation target in the rhythm-based training is cognitive functioning (attention, working memory and executive functions), and secondary targets are motor functions. The module is partly based on the RAS method, which has previously been used in post-stroke motor rehabilitation^25–27^ as well as the Ronnie Gardiner Method used in rhythmic cognitive training after stroke.^24^ In the training videos (altogether 120 videos), a physiotherapist demonstrates a movement or a sequence of movements, which the participant then repeats in the rhythm of the music. The background music has a clear and steady tempo. There are two levels of tempo (slowed and original) and six gross motor movements (placing the hand on the stomach, left or right thigh or shoulder and clapping palms together) and six fine motor movements (eg, grip, pinch). The movements are performed with upper extremities either using left, right or both hands. This enables training with the hemiparetic or both hands, while progressing to more advanced and faster movements.

The rhythm-based training module includes training videos for three different cognitive functions. (1) In training videos targeting maintaining and directing attention, the therapist demonstrates movements that the participant immediately imitates one by one. The movements do not follow a fixed pattern and therefore the participant must maintain attention on the movements throughout the video. (2) In working memory training videos, the therapist demonstrates a sequence of movements that the participant subsequently repeats. The first sequence consists of three movements, and one movement is added with each round (up to a maximum sequence length of nine movements), thereby progressively increasing the working memory span. (3) In training videos targeting executive functions, the participant repeats a sequence of three movements after the therapist. The sequence is altered after two to five rounds without a warning, requiring the participant to adapt to the new sequence, thereby training cognitive control, flexibility and response inhibition.

#### Music listening

The primary rehabilitation target of the music listening module is emotional well-being and secondary targets are cognitive and verbal functions.^33–36^ This module consists of listening to the 20 intervention songs with a model singer and band accompaniment. Each song is available in typical female and male vocal ranges, resulting in 40 videos in total. Participants are instructed to listen to the songs with full attention and to avoid any simultaneous activities. Through listening, participants also familiarise themselves with the intervention songs, thereby supporting the playing-based and singing-based training modules.

#### Music intervention practices

The intervention programme is located on an online platform on a secure web server of the University of Helsinki. The training videos in each module are organised in different categories and are easy to locate in the programme. Participants are provided with a tablet computer with an internet connection, a charger and a loudspeaker, to enable home-based training. Participants receiving playing-based training are also provided with a metallophone. The music therapist provides all participants with brief instructions on how to use the online platform. Participants are also provided with printed instructions with illustrative images. If additional support is required, the instructions may also be provided to a relative, caregiver or another suitable person to assist the participant with the training. The music therapist and trial coordinator provide additional support throughout the intervention period as needed.

The intervention period lasts for 10 weeks. Participants randomised to the intervention group are instructed to train independently for at least 1 hour per day, 5 days per week. The training can be divided into short sessions. Participants in the intervention group have a remote meeting with a music therapist once a week to motivate participants, follow their progress, give feedback and help with possible practical issues. The music therapist tracks participants’ use of the intervention platform throughout the intervention period and uses this information to monitor adherence to intervention protocol and tailor the content of remote sessions accordingly. All remote meetings are delivered by one designated music therapist. The music therapy practices were planned together with a senior music therapist, who also serves as a clinical supervisor and is available as a backup therapist if the primary therapist is unavailable to deliver the remote sessions for any reason. Two remote meetings with the music therapist are recorded to assess treatment fidelity throughout the project.

### Outcome measures

The effectiveness of the music-based telerehabilitation programme is evaluated using tests that measure cognitive functions, aphasia and motor skills (table 1); questionnaires (table 2); structural and functional MRI (s/fMRI); and tasks that are based on the intervention material. Two of the questionnaires (see table 2) are also administered as proxy versions to a family member or caregiver. The outcome measurements are conducted at three time points (T1–T3, figure 1). Parallel versions of memory tests are used across the time points in a randomised order for each participant to minimise learning effects. In addition to the outcome measurements, participants’ musical abilities are assessed at T1 using a comprehensive battery of music tasks.

**Table 1:** Behavioural outcome measures

Primary outcome summary score	Secondary outcome summary score	Measure	Target
Cognitive functioning	Attention	FAT^66^ Trail Making A	Selective attention
		FAT Reaction Time	Selective attention and psychomotor speed
		Simon Task^67^ (congruent)	Selective attention
		SART^68^	Sustained attention
	Working memory	WMS-III^69^ Digit Span	Verbal working memory
		WMS-III Spatial Span	Visuospatial working memory
	Executive function	Simon Task (congruent and incongruent)	Executive function (set-shifting)
		FAT Trail Making A and B	Executive function (set-shifting)
		MoCA^70^ Verbal Fluency (phonemic)	Executive function (cognitive flexibility)
		MoCA	General cognitive function
Speech production		WAB^71^ Conversational Question	Spontaneous speech production
		WAB Picture Description	Spontaneous speech production
		WAB Repetition	Responsive speech production
		WAB Object Naming	Responsive speech production
		WAB Word Fluency (categorical)	Responsive speech production
		WAB Responsive Speech	Responsive speech production
		WAB Sentence Completion	Responsive speech production
Upper-extremity motor function		Action Research Arm Test^72^	Upper-extremity motor function
		Box and Block Test^73^	Gross manual dexterity
		Purdue Pegboard Test^74^	Fine manual dexterity
Not included in a primary or secondary outcome summary score		WMS-III Immediate and Delayed Word List	Verbal memory and learning
		RBMT^75^ Immediate and Delayed Story Recall	Episodic memory
		BIT^76^ Star cancellation	Unilateral visuospatial neglect
		WAB Sequential Commands	Verbal comprehension
		BDAE^77^ Articulatory Agility	Motor production of speech
		BDAE Automatised Sequences	Non-propositional speech

BDAE, Boston Diagnostic Aphasia Examination; BIT, Behavioural Inattention Test; FAT, Flexible Attention Test; MoCA, Montreal Cognitive Assessment; RBMT, Rivermead Behavioural Memory Test; SART, Sustained Attention to Response Task; WAB, Western Aphasia Battery; WMS-III, Wechsler Memory Scale Third Edition.

**Table 2:** Self-report outcome measures

Primary outcome summary score	Measure	Target
Mood and quality of life	PROMIS^78^ Depression	Depressive symptoms
	PROMIS Anxiety	Anxiety symptoms
	Stroke Impact Scale*^79^	Overall quality of life after stroke
Not included in a primary or secondary outcome summary score	Motor Activity Log^80^	Severity of upper-extremity paresis
	Communicative Activity Log*^81^	Quality and quantity of communication in daily life
	Cognitive Failures Questionnaire^82^	Severity of attention and executive deficits in daily life
	Prospective and Retrospective Memory Questionnaire^83^	Memory functioning in daily life

* Also administered as proxy versions to a family member or caregiver.

PROMIS, Patient-Reported Outcomes Measurement Information System.

All behavioural measurements are carried out in a research laboratory at University of Helsinki and conducted by a psychologist with experience in working with patients who had a stroke. Questionnaires are completed at home and returned to researchers at behavioural measurements. The total duration of the outcome measurements, questionnaires and MRI is approximately 8 hours at T1 and approximately 6 hours at T2 and T3. The measurements are distributed across multiple days at each time point, to ensure that the duration of the measurement sessions remains reasonable for the participants.

#### Primary outcomes

Given the heterogeneity of stroke symptoms and multimodal nature of the intervention, it was considered that no single primary outcome would sufficiently capture all relevant aspects of expected intervention effects. Therefore, four summary scores were defined that represent the main targets of the intervention. The primary outcomes are defined as changes from T1 to T2 in summary scores for cognitive functioning, speech production, upper-extremity motor function and mood and quality of life. The four summary scores are derived from the behavioural tests and questionnaires listed in tables 1 and 2. As the outcome measures are derived from partly different metrics, all scores (total scores, difference scores or reaction/completion times) from behavioural tests and questionnaires are first rescaled to 0–100 range using min-max normalisation:

,$$X_{norm}=100\;\times \;\frac{X-X_{min}}{X_{max}-X_{min}}$$

where X is the original score and $X_{min}$ and $X_{max}$ are the minimum and maximum observed values, respectively, across all participants and measurement time points. For outcome measures in which lower values indicate better performance, the scoring direction is reversed, so that higher values consistently reflect better performance. After normalisation, each summary score is calculated as the mean of the relevant rescaled variables. The cognitive functioning summary score includes tests measuring general cognitive function, sustained attention, working memory and executive functions. The speech production summary score includes the Western Aphasia Battery subtests that measure spontaneous speech production, speech repetition, and naming. The upper-extremity motor function summary score includes motor tests that measure the speed and fluency of gross and fine upper limb movements. Mood and quality of life summary score includes questionnaires that measure subjective mood (depression, anxiety), quality of life, cognitive functioning, communicative skills, and motor functioning after stroke.

#### Secondary outcomes

The secondary outcomes include change in the four primary summary scores from T1 to T3. In addition, the secondary outcomes include change in three domain-specific summary scores (attention, working memory and executive functions; see table 1) from T1 to T2 and T3. Finally, change seen in the test-specific scores from all the behavioural tests and questionnaires from T1 to T2 and T3 are included as secondary outcomes.

#### Intervention tasks

Direct training effects of the intervention are assessed at each measurement time point with a selection of tasks that are practised during the intervention (singing, playing and rhythm-based training). In the singing task, participants sing along to one of the intervention songs with karaoke-style lyrics and a model singer shown on a computer screen. The singing task is audio-recorded to allow analysis of linguistic features (production of the lyrics) and acoustic features (pitch, melody, rhythm). The playing task features the same intervention song as the singing task. Participants perform the song with a metallophone once with each hand. A multitrack audio recording of the metallophone playback session is used to quantify the note onsets, which is then compared with the training video soundtrack (available in Musical Instrument Digital Interface format) to calculate accuracy of the played notes. The performance is also captured using regular video recording and a motion-capture system (Qualisys AB, Göteborg, Sweden) to measure movement quality with eight marker-based motion-capture cameras and two synchronised video cameras. In the rhythm-based training task, participants complete one round of attention-oriented gross and fine motor exercises, performed separately with each hand. The rhythm tasks are recorded using video and motion-capture cameras to measure quality and timing of the movements.

#### Magnetic resonance imaging

The participants undergo s/fMRI at each measurement time point. The MRI scans are acquired at Meilahti Triangle Hospital MRI unit using a Siemens Magnetom Skyra 3T and are performed by an MRI radiographer and an MRI physicist. The sMRI protocol includes three sequences: T1-weighted Magnetisation-Prepared Rapid Acquisition with Gradient Echo (MPRAGE, 5 min), Fluid-Attenuated Inversion Recovery (FLAIR, 5 min) and High Angular Resolution Diffusion-weighted Images (HARDI, 10 min). fMRI consists of T2*-weighted gradient echo planar imaging sequences acquired at rest and during listening to novel and familiar music.^64 65^

### Other measures

#### Musical abilities

The assessment of the musical abilities at T1 is done with a recently developed music task battery created by the Centre of Excellence in Music, Mind, Body and Brain (MMBB) research consortium. The MMBB Musical Battery (MMBB-MB) consists of six sub-batteries. Four of the tasks are performed on a tablet computer and they measure (1) melody and rhythm perception and musical memory; (2) entrainment of hand movements to musical rhythm; (3) singing individual notes, melodies and a familiar song, as well as learning a novel song; and (4) recognition and experience of music-evoked emotions. In addition, (5) spontaneous movement to music, entrainment of whole-body movements to musical rhythm and learning of novel dance movements are assessed using motion capture and video recording. Finally, the MMBB-MB includes a set of questionnaires that are integrated into the background questionnaire in this study. The questionnaires measure (6) music background, genre preferences, reward value of music, use of music in mood regulation, and subjective musical skills.

#### Background variables and intervention engagement

At T1, participants complete a background questionnaire covering basic demographic information (age, handedness, gender, native language, marital status, years of education, occupation), general health status and stroke-related medical history (relevant comorbidities, current medication, use of assistive devices, time and cause of the stroke and details of post-stroke treatment and rehabilitation). The questionnaire also measures engagement in hobbies and recreational activities before and after stroke. In the follow-up questionnaires at T2 and T3, participants report any changes in medication, the rehabilitation they have received and the frequency of their engagement in different hobbies and activities during the follow-up period.

Information about the intervention engagement is collected during and after the intervention. During the intervention, participants are asked to keep a weekly diary in a structured format, in which they record the amount of training completed, the specific exercises performed and any free-form reflections about their training experience. At the end of the intervention, participants complete a feedback questionnaire that assesses the perceived effects of the intervention, meaningfulness and suitability of the exercises, the technical usability of the digital platform and open-ended feedback on strengths and areas for improvement regarding the intervention. In addition, the intervention platform collects log data on the number and length of the daily training sessions, what training videos participants watched, and if they watched the videos until the end.

### Data management

All data are collected and stored at the University of Helsinki according to Finnish Data Protection Act. Only necessary information is collected, and only authorised research staff have access to the data. Data are pseudonymised by assigning every participant an ID number prior to any data collection. Data are labelled with this ID number and stored in a separate location from research documents containing personally identifiable information (research consent, ID decoding key). The data are only used for research purposes and participants will not be recognisable in the reports.

### Data analysis

All analyses will be conducted as both intention-to-treat (ITT) analyses, including participants with missing data at any time point, and per-protocol analyses, by running the ITT analyses on a subsample containing no missing data at any time point and sufficient intervention adherence (completion of at least 50% of the training sessions). The analyses will first be conducted on the full sample, including both participants with left-hemisphere and right-hemisphere stroke, and then separately for the left-hemisphere and right-hemisphere groups. Analyses will be performed using linear mixed-effects models, in which interactions between Group (intervention/control) and Time (T1/T2 or T1/T3) are explored across primary, secondary and other outcome measures. Demographic, musical, and clinical background variables will be summarised in a descriptive table.

### Patient and public involvement

Patient and public involvement has been included throughout the planning process of the study and development of the intervention.

Words and phrases used in the song lyrics of the singing-based training and music listening modules of the intervention were collected directly from persons with aphasia during multiple group discussion sessions held in different cities, organised by the Juttu-tupa network of the Finnish Brain Association. Persons with aphasia identified words, expressions and everyday communication themes that they considered important for everyday life. In addition, they were asked to provide broader themes they considered meaningful that could be used as the thematic contents of the songs. These suggestions guided the creation of the song lyrics so that they would contain useful and meaningful content for participants with aphasia.

The intervention platform and training videos were piloted with people recovering from stroke (five in acute ward and five in outpatient rehabilitation) and with a panel of older adults, including individuals with varying levels of digital literacy and some with cognitive impairment. During the pilot sessions, participants tested the platform both in structured tasks and through free exploration and listened to the first song drafts. The five participants in outpatient rehabilitation also borrowed the intervention equipment and tested the platform independently at home for a week. After testing, the participants evaluated the usability and clarity of the intervention platform, and meaningfulness and difficulty of the exercises. They completed questionnaires with Likert scale and open-ended items and participated in interviews with a researcher. Their feedback led to several modifications, including adjustments to the user interface of the intervention platform (layout, navigation, button clarity, colour-coding), visual design (contrast, colours, font size), and the addition and clarification of instructional videos.

In addition, the selection of outcome measures as well as the design and production of the intervention tasks were carried out in collaboration with healthcare professionals, including a neurologist, neuropsychologist, physiotherapists and music therapists, to ensure clinical relevance and suitability of the outcome measures and intervention for patients who had a stroke.

## Ethics and dissemination

This study has been approved by the Ethics Committee of the Helsinki and Uusimaa Hospital District (decision ID: HUS/5459/2024), and research permits have been obtained from the Helsinki and Uusimaa Hospital District, the Social Services and Health Care Division of City of Helsinki and the Wellbeing Services County of Vantaa and Kerava. The study has been preregistered at ClinicalTrials.gov under the title ‘Efficacy of Music-Based Telerehabilitation After Stroke’ (registration ID: NCT06804304, 3 March 2025). Any important protocol modifications will be reported at the preregistration site.

### Risk management

The intervention was designed to address the needs of patients with stroke, and participation in the intervention may benefit the participants’ recovery. The intervention is expected to be motivating, pleasant and well-tolerated, and there are no known health risks expected. Participants in the control group will also receive the potential benefits of the intervention, as they will have access to the intervention platform after the follow-up period. The intervention is delivered using a non-personal tablet computer lent to the participants by the University of Helsinki and using an online rehabilitation programme. The programme operates with an anonymous identifier, providing security in an unlikely event of a data security breach.

The behavioural measurements and s/fMRI are safe for participants and are carried out by experienced personnel. MRI contraindications are carefully reviewed for each scan using a safety screening form, and the procedure is thoroughly explained to the participant. During scanning, participants have continuous video and audio contact with the staff and may interrupt the scan at any time. All structural MRI images are screened by a neuroradiologist for potential incidental findings, which are communicated to the participant and their medical team with participant’s separate consent.

The outcome measurements lasting 6–8 hours per time point may induce fatigue, stress or discomfort for participants, and all possible efforts are made during the measurements to minimise those effects. The measurements are distributed across several days at each time point, with regular breaks provided during the sessions. After the follow-up period participants can receive verbal feedback regarding their rehabilitation progress based on their performance on the behavioural outcome measurements. The measurements are conducted in accessible facilities, ensuring suitability for participants with mobility limitations. Travel expenses related to measurement sessions are reimbursed.

Taken together, these measures are intended to ensure that the burden of participation remains reasonable and is balanced by the potential benefits of the intervention. Because participation in this study and intervention are expected to have no adverse effects, a data monitoring committee has not been appointed. Any adverse effects are however monitored according to protocol. Any negative occurrences that might happen during the behavioural or MRI measurements are reported throughout the data collection. In addition, participants are asked to write down any positive or negative experiences during the intervention in their intervention diary. Participants are insured during the measurements by the University of Helsinki and Helsinki and Uusimaa Hospital District (for MRI conducted at Meilahti Hospital) against unexpected harm. Participation in the study does not affect participants’ usual healthcare or rehabilitation.

### Publication plan

The results will be published in international peer-reviewed scientific journals and in journals for clinical professionals and patient associations in Finland. In addition, the results will be presented in national and international conferences and included in two doctoral dissertations and multiple Master’s theses. All participants will receive a letter informing the main results of the study.

### Relevance and implications for future research and practice

As previous studies have examined different music-based rehabilitation methods independently with narrow target outcomes, this study will fill a gap in the existing literature by providing reliable novel information about the benefits and efficacy of multicomponent music-based rehabilitation for patients who had a stroke. Furthermore, this study aims to clarify the underlying neural and behavioural mechanisms as well as individual factors that contribute to the efficacy of such rehabilitation. The study will also offer valuable insights on the implementation and feasibility of music-based rehabilitation programmes delivered in a remote format for clinical populations. These findings may inspire new avenues for research and support implementation of multicomponent telerehabilitation programmes.

The intervention programme was designed to be scalable and transferable to public rehabilitation platforms, with the aim of making the programme freely available to all patients with stroke in Finland on completion of the study. The results of this study may initiate changes and new practices in stroke rehabilitation and, in the end, improve the accessibility and availability of rehabilitation for patients who would otherwise have less or no rehabilitation at all. The development and evaluation of novel, cost-effective practices are increasingly important given the growing number of people affected by stroke.
