# Supplementary material for: Efficacy of music-based telerehabilitation after stroke: protocol description of a randomised controlled trial
Source: BMJ Open. 2026 Sep 24;16(9):e124110. doi: 10.1136/bmjopen-2026-124110 (PMC13630051; doi:10.1136/bmjopen-2026-124110)
Supplement: Supplementary file 1 [file bmjopen-2026-124110.reviewer_comments.pdf]

PEER REVIEW HISTORY

BMJ Open publishes all reviews undertaken for accepted manuscripts. Reviewers are asked to complete a checklist review form and are provided with free text boxes to elaborate on their assessment. These free text comments are reproduced below.

ARTICLE DETAILS

Title (Provisional)

Efficacy of music-based telerehabilitation after stroke – protocol description of a randomised controlled trial

Authors

Laakso, Ella; Oksanen, Emma; Kaila, Lotta Maaria; Salakka, Ilja; Kauramäki, Jaakko; Lahdenperä, Netta; Pitkäniemi, Anni; Sihvonen, Aleksi J.; Moisseinen, Nella; Särkämö, Teppo

---

VERSION 1 - REVIEW

**Reviewer**	**1**
**Name**	**Tamplin, Jeanette**
**Affiliation**	**The University of Melbourne, Music Therapy**
**Date**	**17-Jul-2026**
**COI**	**None**

Thanks for the opportunity to review this manuscript describing a protocol for a post-stroke music-based telerehabilitation RCT. The article is very well written, and it is great to see such a well-designed and powered study in this area. The detail presented about the music intervention is also commendable.

I have just a few questions about the design and statistical analysis plan.

With your stratification for lesion location, time since stroke, and stroke severity, are you planning subgroup analyses or is this stratification only for the purpose of ensuring balanced allocation?

Most clinical trials have a single prespecified primary outcome. This directly measures the main objective and determines the study's sample size. Having only one primary outcome prevents the inflation of false-positive (Type I) statistical errors. A single prespecified outcome can provide a definitive answer to the trial's central question. How do you plan to interpret your study findings if some outcomes improve and others don’t?

What is the research question (or questions) that you are aiming to answer through this study? In the “Objectives and Research Questions” section you list 4 objectives but no research questions.

The use of composite outcomes or “summary scores” by combining multiple endpoints into a single variable will require careful statistical handling to prevent misleading results. What do you mean by “min-max normalisation” in relation to rescaling all scores to 0-100? Do you have a reference for another study that uses this composite outcomes approach?

Are you measuring the intervention tasks to assess direct training effects at all timepoints?

Line 498 has a typo – should be “also

engagement…”

Line 609 also has a minor expression error – should be “write down any positive or negative experiences during the intervention

their intervention diary”

**Reviewer**	**2**
**Name**	**Lo, Temmy**
**Affiliation**	**Hong Kong Baptist University**
**Date**	**20-Jul-2026**
**COI**	**None**

Thank you for the opportunity to review this well-written trial protocol. I have a few minor suggestions that may help further strengthen the manuscript.

Introduction

- Lines 88–89: The authors state that rehabilitation services in public healthcare settings are often insufficient. It would be helpful to further elaborate on the specific gaps between existing services and the needs of stroke survivors.

- The rationale for selecting singing-based, playing-based, and rhythmic-based interventions could be further clarified. In particular, why are all participants required to receive rhythmic-based and music listening interventions, rather than evaluating these components separately (e.g., through a multi-arm or factorial design) to isolate their individual effects?

- The study hypotheses should be explicitly stated.

Methods

- The inclusion criteria require further clarification. How are “mild impairments” operationally defined? The statement that “participants with TIA or minor stroke may be included” is vague. Please specify whether such participants will be included and under what

criteria. In addition, it should be clarified whether the study includes only first-ever stroke cases or also recurrent strokes.

- More detail is needed regarding how treatment fidelity will be ensured and assessed (e.g., training, monitoring, adherence measures).

Administrative issues

- For transparency, please specify the role of the funder in accordance with SPIRIT checklist item 3c.

VERSION 1 - AUTHOR RESPONSE

Reviewer 1

Dr. Jeanette Tamplin
The University of Melbourne
Comments to the Author:
Thanks for the opportunity to review this manuscript describing a protocol for a post-stroke music-based telerehabilitation RCT. The article is very well written, and it is great to see such a well-designed and powered study in this area. The detail presented about the music intervention is also commendable. I have just a few questions about the design and statistical analysis plan.

> **Reply:** Thank you for the positive and constructive feedback and your insightful comments.

Comment 1: With your stratification for lesion location, time since stroke, and stroke severity, are you planning subgroup analyses or is this stratification only for the purpose of ensuring balanced allocation?

> **Reply 1:** The stratification for lesion location, time since stroke, and stroke severity is indeed used to ensure balanced allocation of patients across the intervention and control groups. As outlined in section 'Data analysis', the statistical analyses will first be conducted on the full sample, including both participants with left- and right-hemisphere stroke, and then separately for the left- and right-hemisphere groups.

Comment 2: Most clinical trials have a single prespecified primary outcome. This directly measures the main objective and determines the study's sample size. Having only one primary outcome prevents the inflation of false-positive (Type I) statistical errors. A single prespecified outcome can provide a definitive answer to the trial's central question. How do you plan to interpret your study findings if some outcomes improve and others don't?

> **Reply 2:** We acknowledge that a single prespecified primary outcome is often preferable, particularly when study is designed to address a narrowly defined research question. However, in the present study the intervention is purposefully designed to support recovery on multiple domains of functioning, and it is further tailored to the most functionally relevant symptomology according to lesion side. Consequently, the relevant rehabilitation targets differ between participants. We

therefore concluded that no single outcome measure would fully capture the overall effectiveness of the intervention, whereas a single overarching composite score would incorporate areas that were not rehabilitated in all participants. To address this challenge, we defined four domain-specific summary scores that represent the main rehabilitation target areas: cognitive functioning, speech production, upper-extremity motor function, and mood and quality of life. To clarify this in the manuscript, we added a statement at the beginning of 'Primary outcomes' section explaining the rationale for including multiple primary outcomes (page 15, lines 429-432). We also acknowledge that improvements may occur in some areas but not in others, and it might even be expected for the domains that are not rehabilitated in each participant group. To facilitate transparency and interpretation, we will report both the summary scores and their individual components. Furthermore, appropriate corrections for multiple comparisons will be applied where relevant to minimise the risk of inflated Type I error.

Comment 3: What is the research question (or questions) that you are aiming to answer through this study? In the "Objectives and Research Questions" section you list 4 objectives but no research questions.

Reply 3: We agree that the 'Objectives and Research Questions' section required clarification. The section has now been revised to explicitly state the research questions and hypotheses (page 6, lines 159-167).

Comment 4: The use of composite outcomes or "summary scores" by combining multiple endpoints into a single variable will require careful statistical handling to prevent misleading results. What do you mean by "min-max normalisation" in relation to rescaling all scores to 0-100? Do you have a reference for another study that uses this composite outcomes approach?

Reply 4: We agree that the construction and interpretation of summary scores require careful consideration. The rationale for using summary (or composite) scores pooled from individual test/questionnaire scores is that they can potentially provide more reliable estimates of the underlying domains measured by individual tests/questionnaires as well as capture broader functional outcomes. We have previously used a similar approach in music intervention studies in stroke patients (Särkämö et al. 2008 Brain, Sihvonen et al. 2020 Ann Clin Transl Neurol) and persons with dementia (Särkämö et al., 2014 Gerontologist, Särkämö et al., 2016 J Alzheimers Dis). To facilitate interpretation, we will report both summary scores (primary outcomes) and their individual component outcomes (secondary outcomes), which allows readers to evaluate the contribution of the individual measures to any observed effects.

By min-max normalisation, we refer to rescaling each outcome measure to a common 1-100 scale before summation to a composite score. The rescaling is done using the minimum and maximum observed values of each measure (across all patients and time points):

Normalised score = ((score - minimum observed score) / (maximum observed score – minimum observed score)) x 100

We selected this approach because the summary scores include outcome measures that are derived from heterogenous metrics, several of which do not have a meaningful fixed upper limit (verbal fluency, reaction times), which makes standardisation methods based on proportional scores (i.e., percentage of maximum score) unusable. Furthermore, for some measures with a bounded theoretical range, the observed score range will likely represent only a small portion of the theoretical range (eg, error counts or memory task total scores). To improve transparency, we have now clarified the normalisation procedure in the revised manuscript (page 15, lines 436-445).

Comment 5: Are you measuring the intervention tasks to assess direct training effects at all timepoints?

Reply 5: The intervention tasks are indeed performed at all time points to evaluate the direct training effects over the intervention period (T1-T2) and follow-up period (T1-T3). We have now clarified this in the revised manuscript by highlighting this information also on the beginning of *Intervention tasks* section (page 16, line 461).

Comment 6: Line 498 has a typo – should be "also measures engagement…"

Reply 6: This typo is now corrected in the revised manuscript (page 17, line 509).

Comment 7: Line 609 also has a minor expression error – should be "write down any positive or negative experiences during the intervention in their intervention diary"

Reply 7: This expression error is now corrected in the revised manuscript (page 20, lines 616-617.

Reviewer 2

Dr. Temmy Lo, Hong Kong Baptist University

Comments to the Author:
Thank you for the opportunity to review this well-written trial protocol. I have a few minor suggestions that may help further strengthen the manuscript.

Reply: Thank you for the positive and constructive feedback and your insightful comments.

Comment 1: Lines 88–89: The authors state that rehabilitation services in public healthcare settings are often insufficient. It would be helpful to further elaborate on the specific gaps between existing services and the needs of stroke survivors.

Reply 1: We have now revised the Introduction to provide additional information about the needs that remain unmet in the existing services (page 4, lines 84-95).

Comment 2: The rationale for selecting singing-based, playing-based, and rhythmic-based interventions could be further clarified. In particular, why are all participants required to receive rhythmic-based and music listening interventions, rather than evaluating these components separately (e.g., through a multi-arm or factorial design) to isolate their individual effects?

Reply 2: We agree that a clarification of the rationale underlying our study design is needed. Several alternative study designs, including a factorial design where patients would receive the individual components and their varying combinations in different arms, were considered during the planning phase to identify the most appropriate approach. However, we are unfortunately constrained by the relatively small volume of stroke patients within the health care districts we are collaborating with, and recruiting sufficiently large sample for factorial design was not considered practically feasible within the project period. Another option would have been to provide all the intervention components (singing, playing, rhythm-based, music listening) to all patients in the intervention arm, but we were concerned that this could potentially lead to patients feeling overwhelmed by the amount of material or not engaging enough with the components that are not so relevant for their symptom profile (eg, singing for right-hemisphere stroke patients without aphasia). The current design is a compromise between providing patients with sufficient versatility in the type of intervention exercises to facilitate motivation and long-term engagement with the exercises and facilitating training in the domains most affected by their stroke. The importance of maintaining motivation is particularly important in remote rehabilitation where participants practice independently at home; this is also supported in our intervention model by the weekly sessions with the music therapist. The rhythm-based and music listening components are provided to both left-hemisphere and right-hemisphere stroke subgroups because they target cognitive and emotional rehabilitation deficits common to both subgroups. All in all, the multicomponent model reflects the clinical reality of stroke where varying post-deficits often co-occur and would require simultaneous rehabilitation. We have revised the manuscript accordingly to clarify these considerations (page 9, lines 266-274)

Comment 3: The study hypotheses should be explicitly stated.

Reply 3: The study hypotheses have now been added to the 'Objectives and Research questions' section (page 6, lines 164-167).

Comment 4: The inclusion criteria require further clarification. How are "mild impairments" operationally defined? The statement that "participants with TIA or minor stroke may be included" is vague. Please specify whether such participants will be included and under what criteria. In addition, it should be clarified whether the study includes only first-ever stroke cases or also recurrent strokes.

Reply 4: We agree that "mild impairment" was insufficiently defined in the original manuscript. Eligibility is based on the presence of stroke-related symptoms rather than on neuropsychological test result or cut-offs. The symptoms are identified during the recruitment interview and reported by participant, family member, caregiver or healthcare professional. We have now revised the manuscript to provide a more detailed description of the eligibility criteria (page 7, lines 195-199).

Regarding previous strokes, our intention is to exclude participants with pre-existing neurological symptoms that could confound the assessment of stroke-related impairments and intervention effects (eg, by affecting underlying neuroplasticity mechanisms and general capacity for recovery/rehabilitation). The possible occurrence of previous strokes and presence of prior neurological symptoms is screened during the recruitment interview, which is conducted by a member of the research team with knowledge of stroke and its symptomology. To clarify this in the text, we have now revised the eligibility criteria to specify that participants with prior TIA or minor stroke *with no persistent symptoms* can be included (page 7, lines 202-206).

Comment 5: More detail is needed regarding how treatment fidelity will be ensured and assessed (e.g., training, monitoring, adherence measures).

Reply 5: We agree that a more detailed description about the treatment fidelity assessment practices improves the transparency of our study protocol. We have now revised the section to include additional information (pages 12-13, lines 401-406). We now clarify that all of the remote sessions are delivered by one music therapist, which itself enables consistency for the delivery of the intervention. Additionally, a senior music therapist has been involved in the planning of the intervention protocol and the training of the primary music therapist. During the trial implementation, the senior music therapist also provides supervision to the primary music therapist and serves as a backup therapist if needed. In addition, we highlighted that the music therapist has access to the log data collected by the intervention platform and is therefore able to monitor the intervention adherence in real time and tailor the remote session contents accordingly, providing guidance and motivational support to each participant (page 12, lines 399-401). We believe that these procedures effectively address both intervention adherence and consistency of intervention delivery.

Comment 6: For transparency, please specify the role of the funder in accordance with SPIRIT checklist item 3c.

Reply 6: The funders did not have any role in designing, conducting, analysing, or reporting of the trial. This has now been clarified in the Funding statement (page 21, lines 659-660).

VERSION 2 - REVIEW

**Reviewer**	**1**
**Name**	**Tamplin, Jeanette**
**Affiliation**	**The University of Melbourne, Music Therapy**
**Date**	**13-Sep-2026**

COI

Thanks for the clear response to the previous round of review questions. All my concerns have been satisfactorily addressed. All the best with the study!
